# Postoperative outcomes of surgical delay in inflammatory bowel disease patients: a multicenter cohort study

**DOI:** 10.1007/s13304-024-01893-5

**Published:** 2024-05-28

**Authors:** Ellen de Bock, Eline S. Herman, Vincent Meij, Thijs A. Burghgraef, Bas Oldenburg, Paul M. Verheijen, Apollo Pronk, Mando D. Filipe, Menno R. Vriens, Milan C. Richir

**Affiliations:** 1 Department of Surgery, University Medical Center Utrecht, Utrecht, The Netherlands; 2 Department of Gastroenterology and Hepatology, University Medical Center Utrecht, Utrecht, The Netherlands; 3 Department of Surgery, Meander Medical Center, Amersfoort, The Netherlands; 4 Department of Surgery, Diakonessenhuis, Utrecht, The Netherlands

**Keywords:** Inflammatory bowel diseases, COVID-19, Postoperative complications, Surgery

## Abstract

Postponement of surgical inflammatory bowel disease (IBD) care may lead to disease progression. This study aims to determine the influence of delayed surgical IBD procedures on clinical outcomes. This multicenter retrospective cohort study included IBD patients who underwent a surgical procedure during the Coronavirus disease 2019 (COVID-19) pandemic from March 16, 2020, to December 31, 2020, and were compared to a pre-COVID-19 cohort. The primary endpoint was determining the number of (major) postoperative complications. Secondary endpoints were the time interval between surgical indication and performance of the surgical procedure and the risk factors of postoperative complications using multivariate analysis. Eighty-one IBD patients who underwent a surgical procedure were included. The median time interval between surgical indication and performance of the surgical procedure did not differ between the COVID-19 and pre-COVID-19 cohorts (34 vs. 33.5 days, *p* = 0.867). Multivariate analysis revealed a longer time interval between surgical indication and surgical procedure significantly correlated with the risk of developing postoperative complications [odds ratio (OR) 1.03, *p* = 0.034]. Moreover, previous surgery was identified as an independent predictor (OR 4.25, *p* = 0.018) for an increased risk of developing major postoperative complications. There was no significant surgical delay for patients with IBD in the COVID-19 pandemic cohort compared to the pre-pandemic cohort. However, a longer time interval between surgical indication and surgical procedure significantly correlated with the risk of developing postoperative complications. In the event of future scarcity in healthcare, efforts should be made to continue surgical procedures in IBD patients.

**Supplementary Information:**

The online version contains supplementary material available at 10.1007/s13304-024-01893-5.

## Introduction

Patients diagnosed with inflammatory bowel disease (IBD) will likely require a disease-controlling surgical procedure within ten years of diagnosis [1, 2]. Most of these patients are experiencing inadequate control of IBD-specific symptoms, diagnosed with colitis-associated colorectal carcinoma, or presenting with a disease complication such as intestinal bowel obstruction, fistula, perforation, or stenosis [3–5]. Performing surgery on IBD patients may contribute to improve their quality of life (QoL) in the long term [6, 7].

During the Coronavirus disease 2019 (COVID-19), different (surgical) disciplines have taken measures to provide healthcare capacity for COVID-19 patients. Among these measures was the postponement of elective surgical procedures, including those for IBD-related pathologies [8, 9]. In addition, even before the COVID-19 pandemic emerged, the waiting time to perform a surgical IBD procedure in The Netherlands increased [10].

Due to the progressive nature of IBD, postponement of surgical IBD procedures may lead to disease progression, resulting in greater complexity of the surgical procedure and subsequently an increased risk of complications [11, 12].

Currently, studies describing the consequences of delaying surgical IBD-related procedures are limited. This study aims to determine the effect on clinical outcomes of postponed surgical IBD procedures in patients with IBD who underwent a surgical procedure.

## Materials and methods

### Study design and population

This retrospective multicenter cohort study was conducted in three, one academic and two general public hospitals in The Netherlands; University Medical Centre Utrecht, Meander Medical Centre and Diakonessenhuis Hospital. All consecutive patients who underwent a surgical IBD procedure between March 16, 2020, and December 31, 2020, were included and considered the COVID-19 cohort. These patients were compared to patients who underwent a surgical IBD procedure during the same period of the previous year (March 16, 2019, and December 31, 2019) and were defined as the pre-COVID-19 cohort. The start date of the cohorts was in line with the start of the Dutch implemented COVID-19 guidelines to postpone (semi-)elective surgical care [13]. Participants were included if they were older than 18 years and had a pathologically confirmed Crohn’s disease (CD) or Ulcerative Colitis (UC) diagnosis. The ethics committee of all participating centers approved this study and decided that patient-informed consent was not required. The current study was performed in accordance with the STrengthening the Reporting of OBservational studies in Epidemiology (STROBE) guideline [14].

### Endpoints and definitions

The primary endpoint was to determine the number of (major) 30-day postoperative complications. Secondary endpoints were to assess the time interval between the surgical indication date and date of the surgical procedure, the severity of postoperative complications and their associated risk factors. Data was retrospectively extracted from electronic medical records.

To determine the patient’s health status, patients were assessed prior to the surgical procedure using the American Society of Anesthesiologists (ASA) classification [15]. Additionally, the patient’s health status was also stratified in ASA < 3 and ASA ≥ 3. The severity of complications was assessed according to the Clavien–Dindo classification [16]. Major complications were defined as Clavien–Dindo class III or higher.

Surgical procedures included colectomy with ileocolic anastomosis, total colectomy with end ileostomy, proctocolectomy with end ileostomy, proctocolectomy with ileal pouch-anal anastomosis, proctocolectomy, small bowel resection, ileocecal resection, proctectomy, abscess drainage, seton placement, fistulotomy or revision colostomy. Other surgical procedures included creating a colostomy, creating, and removing a pouch and removing neo-terminal ileum. Furthermore, surgical procedures were stratified into acute or elective settings. In addition, surgical procedures were stratified into laparoscopic, open, robotic, or laparoscopic converted to open.

### Statistical analysis

Descriptive characteristics were reported according to the distribution of the data, with continuous parameters noted as medians with interquartile ranges (IQR) or means with standard deviation (SD). Mann–Whitney-*U* test or *t* test were used to test differences between groups of continuous not normally and normally distributed data, respectively. Differences between categorical data were assessed with Chi-square test or Fisher’s exact test. Univariate regression analysis comparing the same operational period before and during the COVID-19 pandemic were performed. Multivariate logistic regression analyses, with a 95% confidence interval, were performed to establish independent predictors, including adjustment of these interacting predictors, of developing (major) complications in patients who underwent a surgical IBD procedure. Two-sided *p* values less than 0.05 were considered statistically significant. All statistical analyses were conducted using Rstudio 1.3.959 (R version: × 64 4.0.2).

## Results

### Baseline characteristics

A total of 81 patients (44 females, 54.3%) underwent a surgical procedure for IBD, comprising 64 (79.0%) CD patients and 17 (21.0%) patients with UC. The mean age of the IBD patients was 31.0 years. In terms of the Montreal Classification, most (39; 48.1%) of the patients received the CD diagnosis between 17 and 40 years (A2). The majority (37, 45.7%) of the CD patients had the ileocolonic disease (L3), and the most common disease behavior was structuring (B2) without perianal disease modifier in 17 (21.0%) patients. For UC patients, the most common disease extend was distal to the left colic flexure (E3) in 9 (11.1%) patients, and most patients (7, 8.6%) were experiencing severe disease (S3) (Supplementary Table 1). The majority (63, 77.8%) of patients were classified ASA 2. In addition, 18 (22.2%) surgical patients were current smokers. Of all included IBD patients, 39 (48.1%) underwent a previous surgical IBD procedure. Most (54.3%) surgical procedures were performed laparoscopically. More than half of the patients (45, 55.6%) used IBD medication during surgical admission, of which 18 (22.2%) patients received corticosteroids (Table 1).

**Table 1:** Baseline table

	COVID-19 *N* = 35	Pre-COVID-19 *N* = 46	Total *N* = 81	*p* value
Mean age, years (SD)	34.6 (19.2)	28.3 (12.9)	31.0 (16.1)	0.102
Sex, *n* (%)				0.826
Female	20 (57.1)	24 (52.2)	44 (54.3)	
Type of IBD, *n* (%)				1
Crohn’s disease	28 (80.0)	36 (78.3)	64 (79)	
Ulcerative colitis	7 (20)	10 (21.7)	17 (21)	
Comorbidity, *n* (%)				1
Yes	22 (47.8)	17 (48.6)	39 (48.1)	
Time between IBD diagnosis and surgical indication, days				0.034
Median [IQR]	1300 [317.5,3007]	3120 [990,5842.8]	2195 [555,4981]	
Time between surgical indication and performance, days				0.867
Median [IQR]	34.0 [7, 66.5]	33.5 [7.3, 66.8]	34.0 [6, 67]	
ASA, *n* (%)				0.557
ASA 1	2 (5.7)	1 (2.2)	3 (3.7)	
ASA 2	27 (77.1)	36 (78.3)	63 (77.8)	
ASA 3	6 (17.1)	8 (17.4)	14 (17.3)	
ASA 4	0 (0)	1 (2.2)	1 (1.2)	
Current smoker, *n* (%)				0.123
Yes	11 (31.4)	7 (15.2)	18 (22.2)	
Unknown	2 (5.7)	2 (4.4)	4 (4.9)	
Surgery setting, *n* (%)				0.353
Elective	25 (71.4)	38 (82.6)	63 (77.8)	
Acute	10 (28.6)	8 (17.4)	18 (22.2)	
Surgical approach, *n* (%)				0.966
Laparoscopic	20 (57.1)	24 (52.2)	44 (54.3)	
Open	9 (25.7)	13 (28.3)	22 (27.2)	
Robotic	1 (2.9)	2 (4.3)	2 (4.3)	
Converted	5 (14.3)	7 (15.2)	12 (14.8)	
Complication, *n* (%)				
≥ 1 complication	22 (62.9)	23 (50.0)	45 (55.6)	0.353
Clavien–Dindo ≥ 3	11 (31.4)	8 (17.4)	19 (23.5)	0.225
IBD medication usage during surgical admission, *n* (%)				0.67
Yes	18 (51.4)	27 (58.7)	45 (55.6)	
Corticosteroids usage during surgical admission, *n* (%)				0.881
Yes	7 (20)	11 (23.9)	18 (22.2)	

*COVID-19* Coronavirus disease 2019, *n* number, *CD* Crohn’s disease, *UC* ulcerative colitis, *SD* Standard deviation, *IQR* interquartile range, *ASA* American Society of Anesthesiology

No significant differences were observed in baseline characteristics between IBD patients who underwent a surgical procedure during the COVID-19 pandemic compared to IBD patients prior to the COVID-19 pandemic. During the pandemic, the number of surgical IBD procedures decreased by 23.9% (46 pre-pandemic vs. 35 pandemic). The median time interval between indication for surgery and performance of the surgical procedure did not differ significantly between the COVID-19 (median 34 days; IQR [7–66.5], *p* = 0.867) and pre-COVID-19 (median 33.5 days; IQR [7.3–66.8]) cohorts. Ileocecal resection was both before and during the pandemic the most performed surgical IBD procedure, comprising 12 (26.1%) and 15 (42.9%) procedures, respectively (Supplementary Table 1). More patients required urgent surgical procedures during the pandemic compared to the pre-pandemic setting (28.6 vs. 17.4%; *p* = 0.353). Of all patients operated on during the pandemic, 22 (62.9%) had one or more postoperative complications compared to 23 (50.0%) patients who underwent the surgical procedure before the pandemic (*p* = 0.353). Major postoperative complications occurred in 11 (31.4%) patients who were operated on during the pandemic compared to 8 (17.4%) patients who were operated on before the pandemic (*p* = 0.225) (Table 1).

### Risk factors for developing overall postoperative complications

Multivariate analysis revealed a statistically significant correlation between a prolonged time interval from surgical indication to performance of the surgical procedure, after adjusting its effect with ASA classification, and an increased risk of developing postoperative complications (OR 1.02; 95% CI 1.004–1.032, *p* = 0.034). Type of IBD, age, ASA ≥ 3 and a previous surgery did not exhibit significant predictive associations with postoperative complications (Table 2).

**Table 2:** Multivariate analysis of risk factors for postoperative complications

Variable	Estimate	OR (95% CI)	Standard error	*z*-value	*p* value
Type of IBD	− 0.200	0.82 (0.236–2.778)	0.619	− 0.323	0.746
Age	0.009	1.01 (0.976–1.043)	0.017	0.510	0.610
Previous surgery	− 0.009	0.99 (0.381–2.570)	0.484	− 0.019	0.985
Time surgical indication to surgical procedure (days)	0.015	1.02 (1.004–1.032)	0.007	2.125	0.034
ASA ≥ 3	− 0.020	0.98 (0.957–1.001)	0.011	− 1.800	0.072

*OR* odds ratio, *CI* confidence interval, *IBD* inflammatory bowel disease, *ASA* American Society of Anesthesiology

### Risk factors for developing major postoperative complications

Multivariate analysis identified a previous surgery as a risk factor significantly correlated with developing major postoperative complications (OR 4.25; 95% CI 1.354–15.608, *p* = 0.018). Other parameters (type of IBD, age, ASA ≥ 3 and time interval between surgical indication and surgical procedure) were not identified as predictors for developing major postoperative complications (Table 3).

**Table 3:** Multivariate analysis of risk factors for major postoperative complications

Variable	Estimate	OR (95% CI)	Standard error	*z*-value	*p* value
Type of IBD	0.482	1.62 (0.414–5.943)	0.668	0.722	0.471
Age	− 0.004	1.00 (0.953–1.039)	0.022	− 0.196	0.845
Previous surgery	1.448	4.25 (1.354–15.608)	0.613	2.362	0.018
Time surgical indication to surgical procedure (days)	0.004	1.00 (0.999–1.012)	0.003	1.275	0.202
ASA ≥ 3	0.001	1.00 (0.979–1.021)	0.010	0.078	0.938

*OR* odds ratio, *CI* confidence interval, *IBD* inflammatory bowel disease, *ASA* American Society of Anesthesiology

## Discussion

This multicenter retrospective cohort analyzed 81 patients with IBD who underwent a surgical procedure for IBD before and during the COVID-19 pandemic. The current study shows a 23.9% reduction in the number of surgical IBD procedures during the pandemic compared to pre-pandemic practice. During the pandemic, 22 (62.9%) patients developed one or more postoperative complications compared to 23 (50.0%) patients who underwent the surgical procedure before the pandemic. Major postoperative complications occurred in 11 (31.4%) patients who were operated on during the pandemic compared to 8 (17.4%) patients who were operated on before the pandemic. The median time interval between the indication for surgery and the performance of the surgical procedure did not differ between the COVID-19 and pre-COVID-19 cohorts. However, multivariate analysis revealed a statistically significant correlation between a prolonged time interval between surgical indication and surgical procedure and an increased risk of developing postoperative complications. Moreover, multivariate analysis identified previous surgery as an independent risk factor significantly correlated with an increased risk of developing major postoperative complications.

The likelihood of requiring a surgical intestinal procedure among IBD patients has decreased over the past six decades [2]. However, a recent study showed that the number of CD patients requiring a surgical procedure remained stable over the last years, despite the introduction of new biologicals [17]. Surgery in patients with IBD procedures has been shown to result in a better quality of life in the long term [6, 7]. Previous literature has already shown that a long diagnostic delay may increase the frequency of adverse clinical outcomes and that these patients more frequently require urgent surgery [11]. Moreover, a recent study showed a lower risk of re-operation in CD patients who underwent surgery early after the IBD diagnosis [18]. However, data on the consequences of delays in surgery are lacking. The risk of future pandemics is plausible and IBD care may also be delayed in times of healthcare scarcity outside of pandemics. Therefore, it is essential to investigate the possible influence of surgical delay on postoperative outcomes in patients with IBD.

The current study shows a 23.9% (46 vs. 35) reduction in the number of surgical procedures in IBD patients during the COVID-19 pandemic compared to the pre-pandemic practice. This may partly be attributed to healthcare institutions that have followed the advice to postpone (semi-)elective surgical procedures where possible [8, 9, 19, 20]. In addition, this decrease may be due to the fact that patients themselves have chosen to postpone the surgical procedure for fear of contracting COVID-19 in the hospital. This may lead to disease progression, with the potential to negatively affect the perceived quality of life of IBD patients [21, 22].

During the pandemic, the number of postoperative complications increased (62.9 vs. 50%) although not statistically significant (*p* = 0.353). This increase is in line with a previous study demonstrating no significant difference in complications following clinical colorectal resections during the COVID-19 pandemic [23].

Multivariate analysis showed that the time interval between the surgical indication and the surgical procedure was, after adjusting the interaction with ASA classification, significantly correlated (OR 1.02; 95% CI 1.004–1.032, *p* = 0.034) with developing postoperative complications. The association between surgical delay and postoperative complications has been reported in earlier literature, with increased infection and mortality rates after the delay of elective surgical colorectal procedures [24]. In addition, multivariate analysis identified that a previous surgery was a significantly independent predictor (OR 4.25; 95% CI 1.354–15.608, *p* = 0.018) for the development of major postoperative complications. This is in keeping with a previous study showing that prior resection was a significant risk factor for overall complications after ileocolonic resection for CD patients [25].

This study has several limitations. First, despite being a minority, the current study included 18 (22.2%) patients who underwent an acute surgical IBD procedure, which is not suitable for delay. Therefore, future research should focus on the inclusion of elective surgical IBD procedures only. Second, our COVID-19 cohort starts after the implementation of the viral-reducing-measures and ends in December 2020. As a result, a potential catch-up in surgical IBD procedures after the first wave may have potentially been enrolled in our COVID-19 cohort. Hence, a reliable assessment of the first wave’s effect on the number of surgical IBD procedures cannot be made. However, this study shows that, despite possible surgical catch-up procedures, fewer operations were performed during the pandemic. Third, our study includes patients with IBD who underwent various surgical procedures. Therefore, drawing conclusions for specific procedures is difficult. However, our study provides an overarching perspective on current surgical IBD practice. Finally, the present study focused on surgical care, although measures were also implemented for non-surgical IBD care [19, 20]. Since IBD is non-curable and carries a considerable morbidity, additional research to prevent further deterioration of quality of life is paramount.

## Conclusion

The current study shows that the number of surgical procedures in patients with IBD decreased during the COVID-19 pandemic. Multivariate analysis showed that a longer time interval between surgical indication and surgical procedure significantly correlated with the risk of developing postoperative complications. Moreover, a previous surgical procedure was identified as a risk factor for an increased risk of developing major postoperative complications. In the event of future scarcity in healthcare resources, the continuation of surgical procedures in patients with IBD should be pursued since postponement may result in a higher risk of postoperative complications and associated healthcare resources.

### Supplementary Information

Below is the link to the electronic supplementary material.

Supplementary file1 (DOCX 19 KB)

## Data Availability

Research data is available from the corresponding author upon reasonable request.
