# Supplementary material for: Postoperative outcomes of surgical delay in inflammatory bowel disease patients: a multicenter cohort study
Source: Updates Surg. 2024 May 28;76(4):1271–7. doi: 10.1007/s13304-024-01893-5 (PMC11341736; doi:10.1007/s13304-024-01893-5)
Supplement: Supplementary file 1 — Supplementary file1 (DOCX 19 KB) [file 13304_2024_1893_MOESM1_ESM.docx]

Supplementary table 1. Details regarding disease stage and surgical procedures performed on the included patients

|  | COVID-19  N=35 | Pre-COVID-19  N=46 | Total  N=81 | P-value |
| --- | --- | --- | --- | --- |
| **Mean age, years (SD)** | \| 34.6 (19.2) \| \| --- \| | 28.3 (12.9) | 31.0 (16.1) | 0.102 |
| **CD age at diagnosis,** n (%) |  |  |  | 0.195 |
| A1 (≤ 16) | 4 (11.4) | 5 (10.9) | 9 (11.1) |  |
| A2 (17-40) | 14 (40.0) | 25 (54.3) | 39 (48.1) |  |
| A3 (> 40) | 10 (28.6) | 6 (13.0) | 16 (19.8) |  |
| **CD disease location,** n (%) |  |  |  | NA |
| L1 (ileal) | 8 (22.9) | 7 (15.2) | 15 (18.5) |  |
| L2 (colonic) | 3 (8.6) | 6 (13.0) | 9 (11.1) |  |
| L3 (ileocolonic) | 15 (42.9) | 22 (47.8) | 37 (45.7) |  |
| L4 (upper GI) | 0 (0) | 0 (0) | 0 (0) |  |
| L1+L4 | 1 (2.9) | 0 (0) | 1 (1.2) |  |
| L2+L4 | 0 (0) | 0 (0) | 0 (0) |  |
| L3+L4 | 1 (2.9) | 1 (2.2) | 2 (2.5) |  |
| **CD disease behaviour,** n (%) |  |  |  | 0.0543 |
| B1 non-stricturing, non-penetrating | 6 (17.1) | 3 (6.5) | 9 (11.1) |  |
| B2 stricturing | 8 (22.9) | 9 (19.6) | 17 (21.0) |  |
| B3 penetrating | 5 (14.3) | 7 (15.2) | 12 (14.8) |  |
| p perianal disease modifier | 0 (0) | 7 (15.2) | 7 (8.6) |  |
| B1 + p perianal disease modifier | 4 (11.4) | 2 (4.3) | 6 (7.4) |  |
| B2 + p perianal disease modifier | 0 (0) | 4 (8.7) | 4 (4.9) |  |
| B3 + p perianal disease modifier | 5 (14.3) | 4 (8.7) | 9 (11.1) |  |
| **UC disease extent,** n (%) |  |  |  | NA |
| E1 limited to rectum | 0 (0) | 0 (0) | 0 (0) |  |
| E2 proximal | 4 (11.4) | 3 (6.5) | 7 (8.6) |  |
| E3 distal to flexura lienalis | 3 (8.6) | 6 (13.0) | 9 (11.1) |  |
| **UC disease severity,** n (%) |  |  |  | 0.416 |
| S0 remission/asymptomatic | 1 (2.9) | 0 (0) | 1 (1.2) |  |
| S1 mild | 2 (5.7) | 1 (2.2) | 3 (3.7) |  |
| S2 moderate | 2 (5.7) | 4 (8.7) | 6 (7.4) |  |
| S3 severe | 2 (5.7) | 5 (10.9) | 7 (8.6) |  |
| **Type of IBD surgery,** n (%) |  |  |  | 0.344 |
|  |  |  |  |  |
| Colectomy with ileocolic anastomosis | 1 (2.9) | 1 (2.2) | 2 (2.5) |  |
| Colectomy with end ileostomy | 2 (5.7) | 4 (8.7) | 6 (7.4) |  |
| Proctocolectomy with end ileostomy | 2 (5.7) | 0 (0) | 2 (2.5) |  |
| Proctocolectomy with ileal pouch-anal anastomosis | 1 (2.9) | 4 (8.7) | 5 (6.2) |  |
| Proctocolectomy | 0 (0) | 1 (2.2) | 1 (1.2) |  |
| Small bowel resection | 3 (8.6) | 2 (4.3) | 5 (6.2) |  |
| Ileocecal resection | 15 (42.9) | 12 (26.1) | 27 (33.3) |  |
| Proctectomy | 3 (8.6) | 4 (8.7) | 7 (8.6) |  |
| Abscess drainage | 3 (8.6) | 1 (2.2) | 4 (4.9) |  |
| Seton placement | 2 (5.7) | 5 (10.9) | 7 (8.6) |  |
| Fistulotomy | 2 (5.7) | 4 (8.7) | 6 (7.4) |  |
| Revision colostomy | 0 (0) | 1 (2.2) | 1 (1.2) |  |
| Other | 1 (2.9) | 7 (15.2) | 8 (9.9) |  |

COVID-19 = Coronavirus Disease 2019, n = number, CD = Crohn’s disease, UC = Ulcerative Colitis, SD = Standard deviation, ASA = American Society of Anesthesiology.
